# Fulminant Form of Guillain-Barré Syndrome Complicated by Hematoma of the Corpus Callosum Occurring in the Context of Head Trauma: A Case Report

**DOI:** 10.7759/cureus.77919

**Published:** 2025-01-24

**Authors:** Manira Moussa Ahmed, Mahamoud Ahmed Houssein, Jihane Ziati, Nezha Dini, Amal Haoudar

**Affiliations:** 1 Pediatrics, Cheikh Khalifa International University Hospital, Mohammed VI University of Health Sciences, Casablanca, MAR; 2 Anesthesiology and Reanimation, Cheikh Khalifa International University Hospital, Mohammed VI University of Health Sciences, Casablanca, MAR; 3 Anesthesia and Critical Care, Cheikh Khalifa International University Hospital, Mohammed VI University of Health Sciences, Casablanca, MAR; 4 Paediatrics, Faculty of Medicine and Pharmacy of Rabat, Cheikh Khalifa International University Hospital, Mohammed VI University of Health Sciences, Casablanca, MAR

**Keywords:** artificial ventilation, axonal sensorimotor neuropathy, dysautonomic crisis, guillain barre’s syndrome (gbs), head trauma

## Abstract

Guillain-Barré syndrome (GBS) is an acute, demyelinating, immune-mediated polyradiculoneuropathy, often triggered by an infection. It is the most common cause of acute flaccid areflexic paralysis in children. Although generally associated with infections, this article presents a rare case with a rapid onset, where GBS was revealed following a context of head trauma. A 2.5-year-old boy, with no significant medical history, was admitted to the intensive care unit for severe acute respiratory distress, occurring 48 hours after a head trauma. Clinical examination revealed severe dyspnea without fever, and tetraparesis on admission, progressing to tetraplegia during his stay in the intensive care unit, with sensory deficit and abolished deep tendon reflexes, followed by peripheral facial diplegia. Imaging studies were normal, and cerebrospinal fluid analysis showed a characteristic albuminocytologic dissociation, typical of GBS, with no signs of meningitis. The patient required intubation and mechanical ventilation. The diagnosis of GBS was confirmed by an electroneuromyogram (ENMG), showing signs of severe sensory-motor axonal polyradiculoneuropathy. Intravenous immunoglobulin (IVIg) therapy was administered, and the patient's condition gradually improved, leading to extubation after 20 days of respiratory support. The risk factors for respiratory failure and biological markers, such as lymphopenia, are also discussed in this case report. Although GBS is a rare pediatric neurological emergency, this case illustrates how the condition can mimic other pathologies and occur in the context of head trauma, particularly in cases with axonal involvement. This can lead to a diagnostic delay. This article emphasizes the importance of early diagnosis to improve the vital prognosis and reduce the mortality associated with this potentially severe condition.

## Introduction

Guillain-Barré syndrome (GBS) was first described in 1859 by Landry and later identified as a distinct nosological entity in 1916 by Guillain, Barré, and Strohl, with the advent of lumbar puncture and electroneuromyography (ENMG) [1]. It is an acute, immune-mediated, demyelinating polyradiculoneuropathy affecting the peripheral nervous system, often occurring after a post-infectious and post-vaccinal interval [2]. This syndrome is considered the most frequent cause of acute flaccid areflexic paralysis in children, with an incidence of 1 to 2 cases per 100,000 children annually [1,2]. It is characterized by muscle weakness, primarily distal predominant, and may progress to a symmetrical ascending paralysis with absent deep tendon reflexes. In severe cases, it can involve the diaphragm, potentially leading to life-threatening respiratory failure. The positive diagnosis of GBS is based on the clinical presentation and the presence of albuminocytologic dissociation without central nervous system involvement and is confirmed by electroneuromyography [3,4]. Infectious triggers are common in the pathogenesis of GBS. Here, we report a rare pediatric case of fulminant GBS manifesting as acute respiratory distress occurring 48 hours after a head trauma.

## Case presentation

Patient and observation

This is a 2.5-year-old boy, from a non-consanguineous marriage, with no significant medical history except for a flu-like syndrome one week prior to admission. He was admitted to the pediatric intensive care unit for management of severe acute respiratory distress without fever, occurring 48 hours after a head trauma and preceded by bilateral distal-predominant paraparesis.

Clinical description

The patient tripped and fell from a height from the corner of a table at home, which left him with a left fronto-parietal impact, without initial loss of consciousness, seizures, or notable signs of intracranial hypertension. Twenty-four hours later, upon waking, the parents noticed slowness of speech and generalized body malaise, making it difficult to stand, all occurring in the context of afebrile illness. Due to the onset of respiratory distress 48 hours post-trauma, the patient was admitted to the intensive care unit.

Clinical examination

Upon admission, the patient was afebrile, with a Glasgow Coma Scale (GCS) of 13/15 (E: 4 V: 3 M: 6). His capillary blood glucose was normal, with a small left frontoparietal ecchymosis without an obvious wound and no signs of meningeal rigidity. No facial paralysis was noted, but the patient exhibited tetraparesis with absent sensation and absent deep tendon reflexes in all four limbs and a negative Babinski sign bilaterally.

On respiratory examination, the chest was symmetrical, with severe dyspnea, bradypnea, and bronchial congestion, accompanied by crackles on auscultation. The oxygen saturation was 30% on room air. The tracheal suctioning yielded purulent secretions. Hemodynamically, the patient was stable.

A brain CT scan, complemented by a cerebral and spinal MRI, did not show any extradural or subdural hematoma, subacute lesions, or ischemic or parenchymal abnormalities. Cervical slices did not reveal any spinal lesions or cervical fractures. The lumbar puncture showed clear cerebrospinal fluid (CSF), with albuminocytological dissociation, a protein concentration of 0.54 g/L, and a white blood cell count of less than 5/mm³. The CSF culture and viral panel were negative. Table 1 presents the lumbar puncture analysis.

**Table 1:** CSF analysis

Lumbar puncture		
CSF	Clear	
Leukocytes /mm^3^	<5	
Red blood cells /mm^3^	3	
Direct examination	Clear	
CSF glucose levels g/l	0.9	0.4-0.8
CSF protein level g/l	0.54	0.15-0.45
Chloride level mmol/L	119	110-130
Culture	Negative	
Multiplex PCR on CSF	Negative	

**Figure 1:**
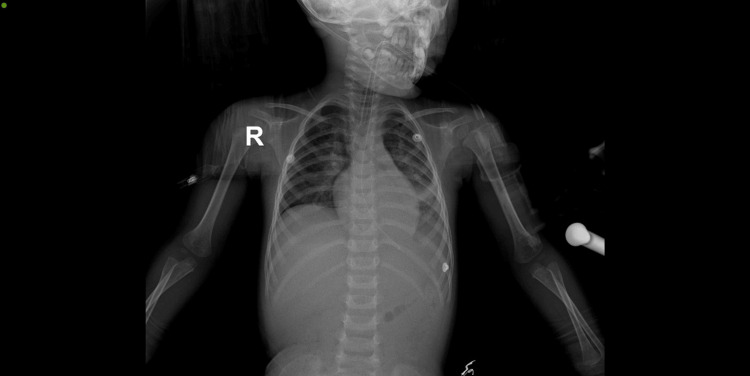
Chest X-ray Bilateral para-hilar opacities more pronounced in the left lung

Chest X-ray revealed a bilateral parahilar pulmonary focus (Figure 1).

Biologically, early arterial blood gas was normal, and there was no evidence of an inflammatory syndrome except an ESR at 48 mm/hr at hour 1. We noted a level of CRP at 6.5, procalcitonin at 0.13, and lymphopenia at 1390 without neutropenia or associated anemia. The lymphocyte subpopulation was notably reduced, with a CD4/CD8 ratio of 1.73. The blood ionogram, renal and liver function tests, and hemostasis were normal. Viral serologies for EBV, CMV, HIV, hepatitis B, C, COVID-19, and Mycoplasma were all negative. A blood culture, protected distal sampling, and urinary and stool cultures were all negative. Additionally, there was a mild elevation of muscle enzymes (CPK 349, LDH 436), with a negative myoglobin. An initial electroencephalogram (EEG) showed no abnormalities. Table 2 summarizes the results of the assessments.

**Table 2:** Summary of the results of the assessments Hb: Hemoglobin, WBC: White blood cells, PNN: Polynuclear neutrophils, CRP: C-reactive protein, SR: Sedimentation rate at 1 hour, ASAT: Aspartate aminotransferase, ALAT: Alanine aminotransferase, PT: Prothrombin, CPK: Creatinine phosphokinase, CBEU: Cytobacteriological examination of the urine

Parameters	Values	Reference values
Hb g/dl	13.1	10.5-13.5
WBC (x10^3 ^cells/ mm^3^)	14.210	6-17.5
PNN (x10^3 ^cells/ mm^3^)	11.610	1 .5-8.5
Lymphocytes (x10^3 ^cells/ mm^3^)	1.390	3-13.5
Monocytes (x10^3^ cells/ mm^3^)	1.180	0.2-1
Platelets (x10^3 ^cells/mm^3^)	354	200-550
CRP mg/l	6.50	<8
PCT ng/ml	0.13	<0.5
Fibrinogen g/L	4.39	2-4.5
SR at H1	48	<13
Sodium mEq/l	141	136-145
Potassium mEq/l	3.5	3.5-5.1
Alkaline reserve mEq/l	15	20-28
Calcium mg/L	102	85-101
Urea g/L	0.38	0.15-0.45
Blood creatinine mg/L	5.10	7-13
ASAT UI/L	31	05-34
ALAT UI/L	17	<55
PT %	85.1%	70-100
Myoglobin ng/mL	33.1	<154.9
CPK UI/l	349	<200
CD3 T lymphocytes (/mm^3^)	29.9 %	2100-6200
	450	
CD 4 T lymphocytes (/mm^3^)	17.3%	1300-3400
	266	
CD 8 T lymphocytes (/mm^3^)	10.0%	620-2 000
	154	
B lymphocytes (CD 19+) (/mm^3^)	32.0%	720-2 600
	493	
NK lymphocytes (CD 16+ CD56+) (/mm^3^)	1.2%	180-920
	18	
CD4/CD8 ratio	1.73	
HIV 1 and 2, CMV, EBV, Herpes 1 and 2, Mycoplasma, COVID 19 serologies were negative		
Blood culture	Sterile	
CBEU	Negative	

Therapeutic decision and evolution

The initial management involved intubation and mechanical ventilation upon admission. A loading dose of phenobarbital (20 mg/kg/day) followed by a maintenance dose (5 mg/kg/day) was immediately initiated to treat possible seizures, with head trauma likely caused by a probable axonal/demyelinating neuropathy. In our case, Guillain Barré syndrome was suspected due to the post-infectious free interval, the distal, bilateral, symmetrical, and ascending sensory-motor deficits preceding the respiratory distress, the albuminocytological dissociation on lumbar puncture, and the normal imaging of the central nervous system. This justified the initiation of intravenous immunoglobulin (IVIg) at 1 g/kg/day for two days. ENMG was difficult to perform initially.

**Figure 2:**
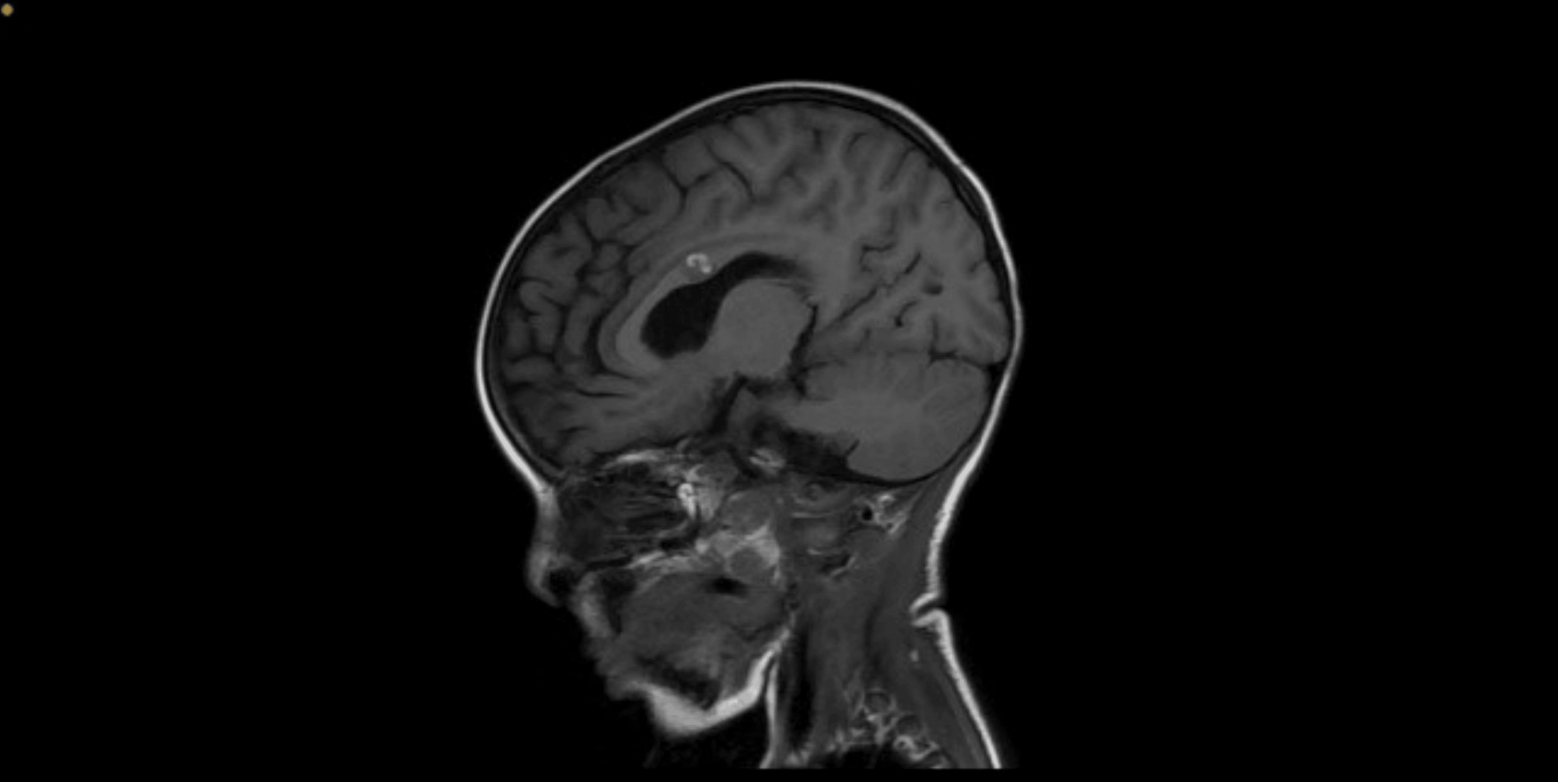
Hematoma of the corpus callosum

The patient, who was minimally sedated, showed no respiratory autonomy upon cessation of sedation on day 7 of hospitalization, with difficult awakening and the onset of systolic hypertension (156/60 mmHg), managed with an intravenous calcium channel blocker (Nicardipine). Then, the neurological examination revealed a comatose patient with a Glasgow scored at 3/15 ( E: 1 V: 1 M: 1), normodilated pupils, bilateral peripheral facial paralysis, tetraplegia, absent sensation and abolished deep tendon reflexes in all four limbs. Given this, a cerebral angio-MRI was performed and revealed a small hematoma in the corpus callosum (Figure 2).

Status epilepticus was noted on a follow-up EEG, which was treated with a second loading dose of phenobarbital and with the maintenance dose initiated on admission.

**Figure 3:**
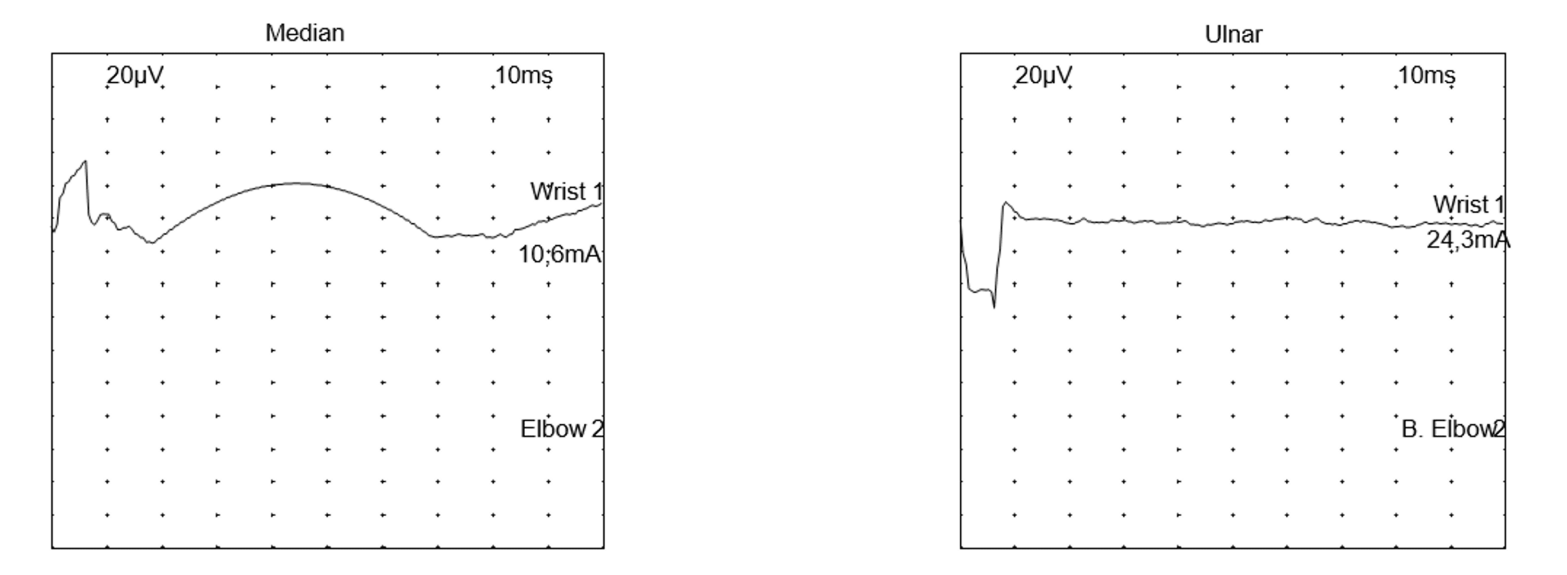
Tracing of sensory conduction of the median and ulnar nerves Stimulation of the median and ulnar nerves revealed no response.

**Figure 4:**
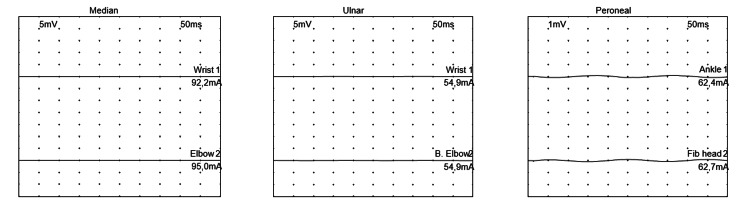
Tracing of motor conduction of the median, ulnar, and peroneal nerves Stimulation of the median, ulnar, and peroneal nerves revealed no response.

ENMG was performed on day 18 of hospitalization (23 days from the onset of his symptoms) and showed the non-excitability of motor and sensory nerves in all four limbs, with positive denervation potentials at rest, consistent with severe sensory-motor axonal polyradiculoneuropathy, thus confirming the diagnosis of Guillain-Barré syndrome (Figure 3 and Figure 4). 

A second course of IVIg was started, this time at 0.5 g/kg/day for 4 days (a total dose of 2 g/kg), with good clinical tolerance. The patient remained on ventilatory support for 20 days and was successfully extubated thereafter. The clinical course was favorable. His stay in the pediatric intensive care unit lasted 34 days, with a total hospital stay of 41 days. At discharge, he had muscle weakness rated at 4, decreased deep tendon reflexes, and was undergoing physical rehabilitation sessions.

## Discussion

The incidence of GBS in the pediatric population is approximately 1 case per 100,000 children per year, with a notable male predominance and a peak incidence at 2 years of age, without any seasonal recurrence, which aligns with our patient's case of a 2.5-year-old male [2,5].

A Danish cohort study conducted over 30 years (1987 to 2016), published in Pediatric Neurology in 2020, collected 125 cases of GBS in children [6]. This study confirms that GBS is the most frequent cause of acute-subacute flaccid areflexic paralysis in children, and no cases of traumatic brain injury were reported. This also highlights the lack of documentation of the context of traumatic brain injury in the literature on GBS [7,8]. The nature of the trauma does not support it as a triggering factor, but rather as a consequential event revealing the pathology. We suggest that the distal motor deficit that began would have caused the traumatic brain injury in our patient.

In two-thirds of cases, the syndrome is preceded by a respiratory or gastrointestinal infection, which is similar to our case, where a recent upper respiratory infection was identified, although no pathogens were isolated in the protected distal sample [9]. The reported infectious episode, purulent pulmonary secretions, and the focal lung finding on the chest radiograph at admission allow us to conclude that the respiratory infection was the triggering factor in our patient's illness. The causative agents most incriminated in the pathogenesis of the disease are Campylobacter jejuni, Mycoplasma pneumoniae, Haemophilus influenzae, HIV, Zika virus, EBV, and CMV [9,10]. This leads to a systemic inflammatory cascade, often referred to as a "cytokine storm". Pro-inflammatory cytokines alter the blood-brain barrier and trigger an autoimmune response by molecular mimicry, targeting the myelin sheath/axon of peripheral nerves. This results in demyelination that leads to conduction blocks in acute inflammatory demyelinating polyradiculoneuropathy (AIDP) and leads to reduced amplitudes in axonopathies. This is consistent with the literature [10].

The diagnosis of Guillain-Barré syndrome is delayed in preschool-age children and the main symptoms are distal paresthesias (50% of cases), muscle weakness of the extremities, and distal neuropathic pain. The demyelinating form is the most common [11]. Table 3 presents the different forms and subforms of Guillain-Barré syndrome.

**Table 3:** Different forms and subforms of Guillain-Barré syndrome IgG: immunoglobulin G

Forms	Frequency	IgG antibodies to
Acute inflammatory demyelinating polyradiculoneuropathy (AIDP)	89–93 %	–
AIDP motor variant		–
AIDP sensory variant		–
Facial diplegia and distal paresthesias	1–5 %	–
Miller-Fisher syndrome (MFS)	5–14 %	GQ1b
Variants of MFS Bickerstaff encephalitis		
Acute ataxic neuropathy		
Acute ophthalmoplegia		
Acute ptosis		
Acute mydriasis		
Acute motor axonal neuropathy (AMAN)		GM1, GD1a
Variants of AMAN acute sensorimotor axonal neuropathy (AMSAN)		GM1, GD1a
Acute motor neuropathy with conduction blocks		GM1, GD1a
Pharyngo-cervico-brachial	1–5 %	GT1a > GQ1b
Paraparetic	2–6 %	GM1 rarely
Acute pandysautonomia	–	Anti-RACh lymph node

The AMSAN form found in our patient is rarer than the AMAN variant and often results in diaphragmatic involvement. It affects children more and is common in Asian countries and Mexico [11,12]. However, the post-infectious interval, the sudden onset of symptoms, the progressive ascending and symmetric flaccid paralysis preceding respiratory signs, swallowing difficulties, and bilateral peripheral facial paralysis correspond to the typical presentation of the disease in the medical literature [12]. However, the rapidity of symptoms (<48 hours) strongly suggests an axonal GBS. Furthermore, our patient met the level 1 diagnostic criteria for GBS according to the Brighton criteria [13].

A study conducted on children with GBS at Necker Hospital in Paris over a seven-year period found that axonal involvement was the most frequent [5]. This form had a more rapid and severe progression, which is consistent with the outcome in our patient who presented with the sensorimotor variant (AMSAN) and experienced a rapidly progressive course. Our patient reached the Nadir in three days and the Hughes score at Nadir was quoted at 5 at admission. Also, the extension phase lasted 3 days and the plateau phase lasted 20 days.

In patients with GBS, predictive factors for the development of respiratory failure during the first week of hospitalization, according to the EGRIS score (Erasmus GBS Respiratory Insufficiency Score), are the time interval between the onset of muscle weakness and patient admission, involvement of facial and/or bulbar muscles, and the Medical Research Council (MRC) sum score [13-15]. The EGRIS score for our patient was 7, indicating an 89% probability of developing acute respiratory failure within the first week of hospitalization. Therefore, an EGRIS score of 4 and above is an indication for mechanical ventilation and ICU admission [15].

The albuminocytological dissociation without pleocytosis is commonly found in GBS, and the ENMG confirms the diagnosis by highlighting the demyelinating polyneuropathy with near-zero conduction velocities, consistent with the data found in the literature [12]. The search for antibodies in the cerebrospinal fluid (CSF), which is costly, was not performed in our case. The immune system is engaged in the demyelating process and the neutrophil/lymphocyte ratio is notably elevated in GBS patients, which emphasizes the nearly constant presence of lymphopenia in these cases. Also, HIV remains a risk factor for a severe form of GBS at all ages. We observed moderate lymphopenia with a collapsed lymphocyte subset in our case [2,12]. Our patient was HIV-negative and the lymphopenia resolved during his stay in the ICU.

Dysautonomic crises, indicative of sympathetic hyperexcitability, occur in 60% of cases during the first week of hospitalization, which was observed in our patient who developed systolic hypertension on day 7 of his hospitalization, further complicated by a small hematoma of the corpus callosum [16-18]. This entity is often seen in post-traumatic cases and is rarely described in the literature concerning GBS [18]. Since the involvement of the corpus callosum is highly epileptogenic, we observed a status epilepticus in our patient, and anticonvulsants were used.

The use of immunoglobulin (IVIg) is the treatment of choice with no evidence of a double course of IVIg in children [12]. Our patient received two courses of IVIg along with antibiotics with favorable clinical progression. Plasmapheresis remains an alternative treatment and has also demonstrated efficacy in GBS, typically requiring four to five sessions of plasma exchange [12,17]. Systemic corticosteroids have not proven effective in this condition, and in our case, we didn't include them. 

A multicenter retrospective study published in 2017, conducted across 15 university hospitals in Japan, collected 177 cases of GBS and emphasized that a high modified ERASMUS score at admission is significantly associated with an inability to walk at 6 months [19]. Our patient's high ERASMUS and EGRIS scores, along with elevated IgG levels at two weeks of disease progression, are poor prognostic markers for GBS in children [20].

## Conclusions

A 2.5-year-old boy, with no significant medical history, was admitted to the intensive care unit for severe acute respiratory distress, occurring 48 hours after a head trauma. A subtle paraparesis was the first clinical symptom of GBS and caused the trauma. The ENMG findings showed severe sensory-motor axonal polyradiculoneuropathy. This peripheral neuropathy is a diagnostic emergency in pediatrics, especially in cases of rapidly progressing axonal involvement. The treatment of GBS in pediatric patients remains IVIg. The particular context of head trauma can lead to diagnostic delays. Through this article, we emphasize the importance of early diagnosis and therapeutic management to reduce the mortality associated with this severe condition in children.
